# Improvement of Triglyceride Levels through the Intake of Enriched-β-Conglycinin Soybean (*Nanahomare*) Revealed in a Randomized, Double-Blind, Placebo-Controlled Study

**DOI:** 10.3390/nu8080491

**Published:** 2016-08-11

**Authors:** Mie Nishimura, Tatsuya Ohkawara, Yuji Sato, Hiroki Satoh, Yoko Takahashi, Makita Hajika, Jun Nishihira

**Affiliations:** 1 Department of Medical Management and Informatics, Hokkaido Information University, Ebetsu, Hokkaido 069-8585, Japan; mnishimura@do-johodai.ac.jp (M.N.); tatuyao@mail.goo.ne.jp (T.O.); ysato@do-johodai.ac.jp (Y.S.); h-satoh@do-johodai.ac.jp (H.S.); 2 Division of Food Function Research, Food Research Institute, NARO, Tsukuba, Ibaraki 305-8642, Japan; youkot@affrc.go.jp; 3 Institute of Crop Science, NARO, Tsukuba, Ibaraki 305-8518, Japan; mhajika@affrc.go.jp

**Keywords:** clinical trial, *Nanahomare*, soybean, triglyceride, β-conglycinin

## Abstract

Soybean is recognized as a beneficial food with various functional components, such as β-conglycinin, which improves lipid metabolism. We evaluated the effects of the β-conglycinin-rich soybean *Nanahomare* on triglyceride (TG) levels. In this randomized, double-blind, placebo-controlled study, we divided 134 adult subjects into test and placebo groups that consumed processed food containing enriched-β-conglycinin soybean or low-β-conglycinin soybean. Hematological tests and body composition measurements were performed at weeks 0 (baseline), 4, 8, and 12 of the study period. TG levels significantly decreased in the test group compared with the placebo group at weeks 4 (change from baseline to week 4, placebo: 0.27 ± 44.13 mg/dL, test: −20.31 ± 43.74 mg/dL, *p* = 0.035) and 12 (change from baseline to week 12, placebo: −0.14 ± 65.83 mg/dL, test: −21.30 ± 46.21 mg/dL, *p* = 0.041). In addition, among subjects whose baseline TG levels were ≥100 mg/dL, the levels significantly improved in the test group at weeks 4 (*p* = 0.010) and 12 (*p* = 0.030), whereas the levels were not different between the test and placebo groups among those whose baseline levels were <100 mg/dL. These results suggest that the ingestion of enriched-β-conglycinin soybean improves serum TG levels.

## 1. Introduction

Coronary heart disease (CHD) is the most common and serious form of cardiovascular disease (CVD) and remains the leading cause of death in Japan. Most CVDs in Japanese people in the 1960s were caused by hypertension; however, in recent years, CVD caused by atherosclerosis has been increasing due to the progressive westernization of the Japanese diet [1]. Japan Atherosclerosis Society Guidelines in 2012 defined the diagnostic criteria for dyslipidemia as low-density lipoprotein cholesterol (LDL-C) ≥ 140 mg/dL, TG ≥ 150 mg/dL and/or high-density lipoprotein cholesterol (HDL-C) < 40 mg/dL to screen subjects with risk factors for atherosclerosis [2]. In order to prevent medical costs from rising in Japan, changing lifestyle for better health, especially dietary habit, is important for improvement of dyslipidemia before beginning medical therapy. Thus, people who want to maintain healthcare by diet pay attention to functional foods, and now much more to many functional foods for prevention of dyslipidemia.

Soybean has been consumed for centuries in many Asian countries, and recently, its popularity has increased markedly in the United States and other Western countries [3]. It has been reported that soybeans, which contain many bioactive components, such as isoflavones and soy protein have health benefits [4,5]. Soybeans significantly lower body mass index (BMI), blood pressure (BP), and serum cholesterol levels in humans [6]. Soybean flavones improve bone metabolism in menopause women via the estrogen-receptor pathway [7]. Soy protein is an important component of soybeans [8] and has a potentially beneficial effect on lipid metabolism [9]. In 1999, the US Food and Drug Administration suggested that intake of 25 g soy protein per day might help reduce the risk of CHD because of decreased serum lipids and lipoproteins [10].

The two major components of soybean protein are glycinin and β-conglycinin [11]. β-conglycinin consists of different combinations of three major subunits, α, α′, and β. Recent in vivo studies have suggested that β-conglycinin can be beneficial for the prevention of various lifestyle-related diseases, including obesity and hypertension [12,13]. Regarding lipid metabolism, it has been reported that β-conglycinin decreased not only serum cholesterol but also triglycerides (TGs) in a hyperlipidemia model rat [14]. In clinical trial, the ingestion of 5.0 g of β-conglycinin suppressed the increase in body fat ratio (BFR) in subjects with a high baseline BFR [15]. Other reports suggested that a significant reduction in visceral fat area was observed after the ingestion of 2.3 g of β-conglycinin [16].

However, large amounts of β-conglycinin (approximately 5 g per day) need to be ingested to perform these functions. This corresponds to approximately 50 to 80 g of soybean per day. Therefore, at present, β-conglycinin is provided as a dietary supplement.

Development and successful production of a new variety of enriched-β-conglycinin soybean has been undertaken by Nagano Research Center of Vegetable and Floricultural Science. Because the amount of β-conglycinin (7S) in soybean is inversely proportional to that of glycinin (11S), decreasing the amounts of 11S successfully led to a corresponding increase in the amounts of 7S [17].

Enriched-β-conglycinin soybean, a new cultivar *Nanahomare*, has been bred by backcrossing Tm-010 (the donor parent of 11S deletion traits) with *Tamahomare* (the recurrent parent), which resulted in the deletion of the transfected 11S. Enriched-β-conglycinin soybean contains approximately 1.8 times more β-conglycinin than ordinary soybeans. This makes it possible to obtain the required amounts of β-conglycinin not only from dietary supplements but also from soybean products, such as soy milk.

In this study, we investigated whether the consumption of enriched-β-conglycinin soybean could improve fasting TG levels in a clinical trial. We designed a double-blind, placebo-controlled study to investigate the effectiveness of enriched-β-conglycinin soybean in decreasing TG levels.

## 2. Materials and Methods

### 2.1. Subjects

We recruited 196 volunteers and selected 149 subjects who had no current medical therapy with high serum triglyceride levels (45 males and 104 females, age range, 30–69 years, TG, 112.01 ± 44.60 mg/dL) through screening, excluding individuals with a recent history of gastrointestinal disorders, pregnancy, severe acute or chronic diseases, surgery, severe allergic reaction to food, particularly soybean, and/or current use of any medication. The clinical intervention was conducted as a double-blind, placebo-controlled trial. At randomization, the 149 eligible subjects were assigned to either the test or placebo group, with adjustments for age, sex, and fasting TG level. The randomization sequence was created using a permuted block randomization design stratified by age, gender, and fasting TG, where the block size was a multiple of 2. Each subject was allocated by third-party data center personnel according to the randomization sequence into the relevant group, thus balancing the numbers in each group. The personnel concealed the allocation information, including the subjects’ personal data, and kept it secured. The information was disclosed only after the laboratory and analysis data were fixed and the method of statistical analysis was finalized.

### 2.2. Study Design

**Figure 1:**
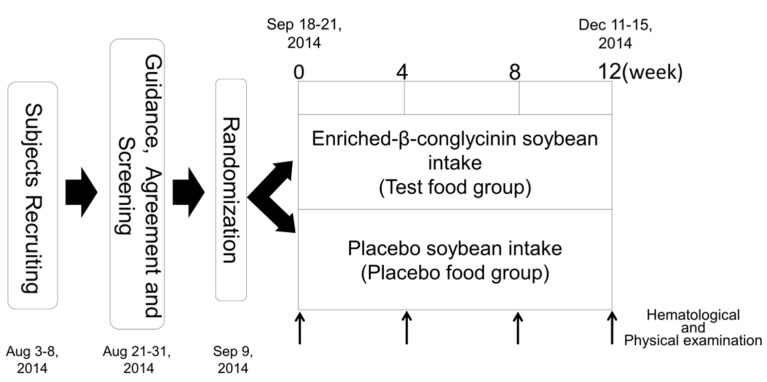
Time schedule (in weeks) for this clinical study. Hematological and physical examinations were conducted at baseline (week 0), and at weeks 4, 8, and 12.

The clinical study was conducted as a double-blind, placebo-controlled trial. The time schedule for the study is shown in Figure 1. We performed body composition measurements, including body weight (BW), BMI, and BFR, at baseline (week 0) and post-intervention at weeks 4, 8, and 12 for the two groups. At all four visits, a medical interview was conducted along with a check of vital signs and hematological examinations. During the course of this study, subjects were asked not to change their daily activities including food consumption and exercise. A medical interview, a check of vital signs, hematological examinations, and body composition measurements were carried out at Hokkaido Information University Center of Health Information Science. The primary outcome was fasting TG. The secondary outcomes were total cholesterol (TC), HDL-C, LDL-C, fasting plasma glucose (FPG), hemoglobin A1c (HbA1c), insulin, BW, BFR, and BMI.

### 2.3. Test Meals

The composition of the soybean products used in this study is given in Table 1. Test soy beans *Nanahomare* were harvested in Nagano, Japan, and placebo soybeans *Nagomimaru* were harvested in Tochigi, Japan. We prepared three kinds of soybean products, namely, flakes (21 g/pack), soy milk (200 mL/pack), and steamed soybean (42 g/pack). We instructed subjects to take 14 packs of soybean products each week, including 7 packs of soybean flakes, 3 packs of soy milk, and 4 packs of steamed soybean. Subjects take 2 packs per day selected from weekly-soybean products (total 14 packs) at favorite time and by favorite cooking methods. However, from week 9 to 12 the subjects took 10 packs of soybean flakes and 4 packs of steamed soybean each week because we could not provide soy milk due to a problem with the manufacturing process. There was no difference in appearance between the test and placebo meals.

The soybean flakes were produced by Asahimatsu Co., Ltd. (Nagano, Japan). The manufacturing process in brief was as follows: after rinsing, the soybeans were soaked in water for a while, followed by a steam sterilization; they were then passed through a drying oven and shaped into flakes. The soy milk samples were produced by Minami Industry Co., Ltd. (Mie, Japan). The manufacturing process in brief was as follows: after drying, the soybeans were dehulled, ground into powder, dissolved in water, and cooked. Steamed soybeans were produced by Oguraya Yanagimoto Co., Ltd. (Hyogo, Japan). Its manufacturing process in brief was as follows: the soybeans were soaked in water with salt and vinegar seasoning for 14 to 20 h and then steamed and sterilized. All the manufacturing plants were quality-controlled in compliance with Japan’s Food Sanitation Act (the Ministry of Health, Labor, and Welfare of Japan). The quantification of soybean β-conglycinin in the processed food products was performed using densitometric methods with the purified β-conglycinin as a reference. Similar results were also obtained using ELISA methods (data not shown). In addition, the presence of each subunit of β-conglycinin in the sample foods was confirmed by western blotting using the specific antibodies raised against purified β-conglycinin.

### 2.4. Physical and Hematological Examination

Blood samples were taken for testing at baseline and at weeks 4, 8, and 12 of the study period. In addition to a medical interview by a doctor, each subject’s body composition (BW, BMI, and BFR) and BP were measured. General blood tests included lipid profile (TG, TC, HDL-C, and LDL-C); blood glucose profile (FPG, HbA1c, and insulin); complete blood count (CBC) this included: white blood cells (WBC), red blood cells (RBC), hemoglobin (Hb), hematocrit (Ht), and platelet count (Plt); liver function, including: aspartate aminotransferase (AST), alanine aminotransferase (ALT), gamma glutamyl transpeptidase (γ-GTP), alkaline phosphatase (ALP), and lactate dehydrogenase (LDH); and renal function: blood urea nitrogen (BUN), creatinine (CRE), and uric acid (UA).

Hematological examinations were performed by Sapporo Clinical Laboratory, Inc. (Sapporo, Japan). Each subject’s body composition and blood pressure were measured with a Body Composition Analyzer DC-320 (Tanita Corp., Tokyo, Japan) and an Automatic Blood Pressure Monitor HEM-7080IC (Omron Colin Co., Ltd., Tokyo, Japan).

### 2.5. Ethics

All subjects provided written informed consent before undergoing any study-related tests, and the study protocol was approved by the ethics committee of Hokkaido Information University (certificate number 2014-07, 23 June 2014). The study protocol was implemented in conformity with the Helsinki Declaration and was registered at the UMIN Clinical Trial Registration System (certificate number UMIN000014427).

### 2.6. Sample Size

The sample size was statistically determined to obtain a power of 80% with an alpha of 0.05. In order to demonstrate effects in TG levels at 12 weeks caused by test meal, which was assumed to give a 20 mg/dL reduction with a standard deviation of 38 mg/dL, a sample size of 120 (60 in the test group and 60 in the placebo group) was required. Assuming a 20% loss to follow-up, 149 subjects were selected.

### 2.7. Statistical Analysis

The means and standard deviations of subject characteristics were calculated for each group. Changes in subject values were analyzed using repeated measures ANOVAs between the groups. In addition, changes in subject values were analyzed using Student’s *t*-test by comparing the means between the test group and the placebo group at each evaluated point. Improvement frequencies of subjects from baseline to week 12 were analyzed using chi-square test between the groups. The primary outcome was determined at week 12. Statistical analyses were performed using SPSS Statistics 19 (IBM, Armonk, NY, USA), and *p* < 0.05 was considered to be significant.

## 3. Results

### 3.1. Subject Dropouts and Exclusions, and Characteristics

**Figure 2:**
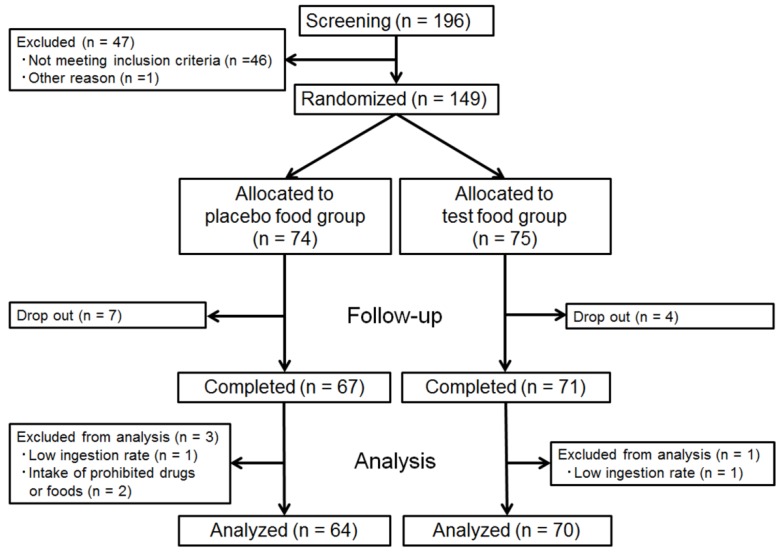
The study flow diagram.

During the trial, 11 subjects dropped out for personal reasons. As a result, 138 subjects completed this trial, 71 in the test group and 67 in the placebo group. Four persons were excluded from analysis because of low ingestion rate (*n* = 2) or intake of prohibited drugs or foods (*n* = 2). As a result, 134 persons (70 in the test group and 64 in the placebo group) were included in the final analysis. The study flow diagram is shown in Figure 2. Mean age, height, BW, BMI, BFR, and fasting TG for each group are presented in Table 2. These data did not differ significantly between the test and placebo groups, indicating the appropriate assignment of subjects into the two groups.

### 3.2. Effect of Enriched-β-conglycinin Soybean on TG

**Figure 3:**
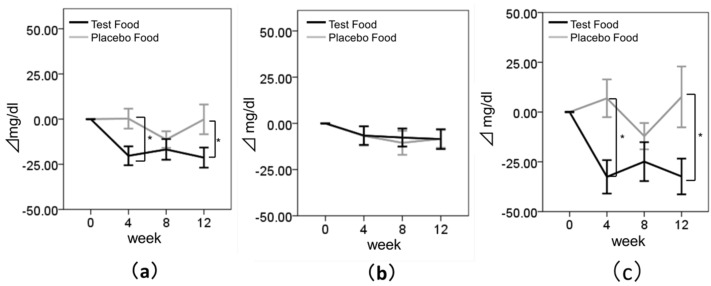
Changes in the levels of TG. Values are means ± SE. (**a**) TG in all subjects; (**b**) TG in subjects whose baseline TG was <100 mg/dL; (**c**) TG in subjects whose baseline TG was ≥100 mg/dL. Black bar, test group; Gray bar, placebo group. * Statistically significant, *p* < 0.05.

First, we evaluated the effect of enriched-β-conglycinin soybean on TG levels (Figure 3 and Table 3). Table 3 shows that the group by time interaction gave difference for TG, though the difference was not statistically significant (*p* = 0.063). In addition, there were no significant differences in the frequencies of improved subjects and unimproved subjects between the test and placebo groups (Table 4). However, there were significant differences between the test and placebo groups in the change in TG from baseline to those at weeks 4 and 12, with the test group showing a marked decrease at each time point (change from baseline to week 4, placebo: 0.27 ± 44.13 mg/dL, test: −20.31 ± 43.74 mg/dL, *p* = 0.035; change from baseline to week 12, placebo: −0.14 ± 65.83 mg/dL, test: −21.30 ± 46.21 mg/dL, *p* = 0.041) (Figure 3a). A previous study suggested that the reduction of TG by intake of purified β-conglycinin was more remarkable in the subjects whose serum TG level was high [18]. To clarify the effects of baseline of TG levels in this study, we divided the subjects into two subgroups with respect to the median of their baseline TG level: a lower-TG group (subjects with TG < 100 mg/dL at baseline; *n* = 64) and a higher-TG group (subjects with TG ≥ 100 mg/dL at baseline; *n* = 70). We judged that this subset analysis was appropriated when compared to the panel of subjects participated in other clinical study dealing with the improvement of serum TG levels by the intake of functional foods [19]. In the lower-TG group, there were no significant differences between the test and placebo groups (Figure 3b). On the other hand, in the higher-TG group, the group by time interaction gave difference for TG, though the difference was not statistically significant (*p* = 0.075), and again, there were significant differences between the test and placebo groups in the change in TG from to those baseline at weeks 4 and 12 (change from baseline to week 4, placebo: 6.91 ± 54.44 mg/dL, test: −32.54 ± 51.03 mg/dL, *p* = 0.010; change from baseline to week 12, placebo: 7.61 ± 87.83 mg/dL, test: −32.35 ± 54.53 mg/dL, *p* = 0.030) (Figure 3c). In addition, there were differences in the frequencies of improved subjects and unimproved subjects between the test and placebo groups, though the difference was not statistically significant (Table 4, *p* = 0.063).

### 3.3. Effect of Enriched-β-Conglycinin Soybean on the Other Lipid Metabolism and Blood Glucose Metabolism Parameters

**Figure 4:**
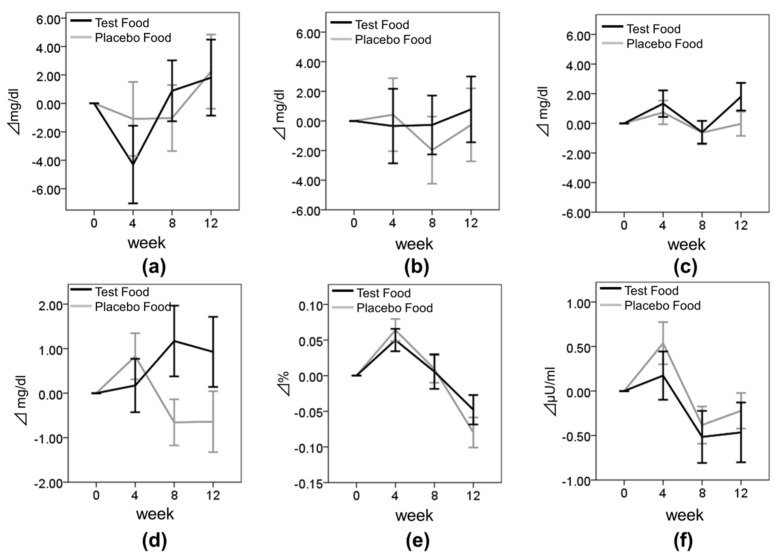
Changes in the lipid metabolism and blood glucose parameters. Values are means ± SE. (**a**) TC; (**b**) LDL-C; (**c**) HDL-C; (**d**) FPG; (**e**) HbA1c; and (**f**) Insulin. Black bar, test group; Gray bar, placebo group.

We also examined the effect of enriched-β-conglycinin soybean on the other lipid metabolism and blood glucose metabolism parameters. We found that the group by time interaction was significant for FPG (*p* = 0.013) (Table 3). However, no significant difference between the groups was observed for FPG at week 12 (Figure 4 and Table 3). The other parameters (TC, LDL-C, HDL-C, HbA1c, and insulin) did not differ significantly between the groups.

### 3.4. Effect of Enriched-β-Conglycinin Soybean on Body Composition

**Figure 5:**
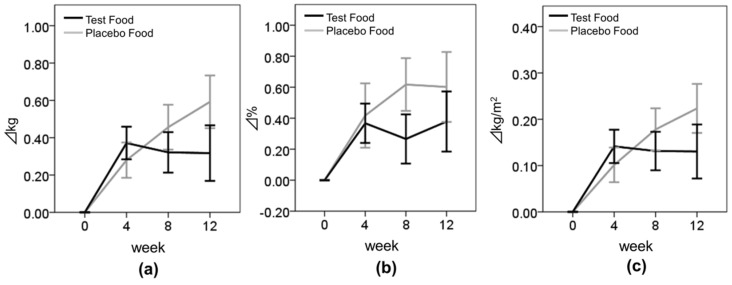
Changes in body composition. Values are means ± standard errors (SEs). (**a**) BW; (**b**) BFR; and (**c**) BMI. Black bar, test group; Gray bar, placebo group.

To confirm the effect of enriched-β-conglycinin soybean on body composition, we evaluated changes in BW, BMI, and BFR. We found that the group by time interaction was significant on BW (*p* = 0.037) (Table 3 and Figure 5). However, the change value between the test group and the placebo group was not difference at each evaluated point.

### 3.5. Levels of Biomarkers of CBC, Liver and Renal Function, BP, and Adverse Events

We examined the levels of several biomarkers of CBC, liver and renal function, and BP after the ingestion of the enriched-β-conglycinin soybean products. As shown in Table 5, minimal changes were observed in the CBC parameters (WBC, RBC, Hb, Ht, and Plt), liver function (ALP, AST, ALT, LDH, and γ-GTP), the biomarkers of renal function (BUN, CRE, and UA), and BP upon arrival. Four subjects showed adverse events (three had dermatosis and one had gastrointestinal disorders), which prevented further participation in the study. The principal investigator judged that two of these dermatosis cases may have been a consequence of the placebo meals and denied relation of other cases to the test and placebo meals. These results suggested that the ingestion of enriched-β-conglycinin soybean had no or minimal unfavorable effects on these parameters even at a dose of 42 g/day and was safe.

## 4. Discussion

The results of our randomized, double-blind, placebo-controlled, parallel-group trial demonstrated the potential effects of enriched-β-conglycinin soybean *Nanahomare* on serum TG levels. TG levels significantly decreased after ingestion of enriched-β-conglycinin soybean for 12 weeks. These results suggest that enriched-β-conglycinin soybean can improve dyslipidemia.

TG levels of the test group decreased significantly compared with those of the placebo group at weeks 4 and 12. This significant decrease was also observed in the subgroups where the subjects had higher baseline TG levels. Mechanisms proposed for how β-conglycinin decreases serum TG levels include the activation of very-low-density-lipoprotein receptors [20], inhibition of fatty acid synthesis in the liver, activation of fatty acid oxidation [21], and inhibition of lipid absorption from the small intestine [22]. A previous study has reported that the ingestion of candy containing β-conglycinin for four weeks decreased serum TG levels in subjects with a baseline TG level > 1.69 mmol/L, and that it decreased visceral fat and BFRs in human subjects [18]. Our clinical study suggested that the enriched-β-conglycinin soybean *Nanahomare* had the potential effect to improve serum TG levels similar to the purified β-conglycinin in dietary supplement. Moreover, the subjects in our clinical trial were mixed in males and females. Previous report suggested that the effects of dietary intervention were different between genders [23]. However, no differences in the reduction of TG level by ingestion of enriched-β-conglycinin soybean were observed between men and women in our clinical trial. In addition, TG level was substantially decreased at week 8 but it was elevated at week 12 in the placebo group. Placebo soybean also contained various functional components such as soy protein, fiber, and isoflavone [4,5], therefore these components might decrease TG levels in placebo group at week 8. However, the effectiveness of TG reduction by ingestion of placebo soybean was observed dissipating at week 12. These results might suggest that β-conglycinin had more large effectiveness of TG reduction than other components of soybean.

Previous reports demonstrated that the TG and visceral fat lowering-effect of β-conglycinin was based on the improvement of insulin resistance in vivo [12]. However, in our clinical trial, the FPG of the test group tended to increase compared with the placebo group at week 8 (change in FPG from baseline to week 8, placebo: −0.66 ± 4.15 mg/dL, test: 4.15 ± 1.17 mg/dL, *p* = 0.060). Although the reason of this was unclear, FPG showed normal levels in most of the subjects during our clinical study. Therefore underlying glucose metabolism improvement by test meal intake may have not been detected to the subjects with normal FPG value. In addition, no differences in other blood metabolism parameters such as HbA1c and insulin were observed between the test meal group and placebo meal group. We need further investigation of the effects of the test meal on blood glucose metabolism against dysglycemia.

Body composition parameters—BW, BMI, and BFR—tended to increase during the test period in both groups. As this study was conducted between September and December, i.e., from fall to winter, physical activities may have decreased because of the cold weather and snow. In spite of possible seasonal effects, the increase of the body composition seemed to be suppressed in the test group. Moreover, BMI tended to be suppressed more in the higher-TG subgroup of the test group than in the placebo group (*p* = 0.098). In addition, the change in the logarithmically transformed TG values from baseline to week 12 was highly correlated with the change in BMI values from baseline to week 12 in the test group but not in the placebo group (Pearson correlations: placebo group, *p* = 0.514; test group, *p* = 0.020) (Figure S1). Supplementation with tea catechins significantly reduced visceral and liver fat accumulation in obese-model mice, and increased β-oxidation activity and the mRNA expression of β-oxidation-related enzyme in the liver [24]. In addition, green tea extract rich in catechin polyphenols and caffeine increased energy expenditure via sympathetic activation of thermogenesis and promoted fat oxidation in humans [25]. β-conglycinin also suppressed fatty acid synthesis and activated fatty acid oxidation in the liver [22]. These findings suggested that enriched-β-conglycinin soybean decreased body composition and serum TG by activating fatty acid oxidation in the liver. Moreover, Kohno et al. reported that the intake of 2.4 g β-conglycinin for 12 weeks decreased the visceral fat area in 30 subjects with BMI ≥ 25 kg/m^2^ [18]. In our study, there were few obese subjects (BMI ≥ 25 kg/m^2^), therefore the subjects who were not obese may have not achieved body fat reduction significantly by test meal intake. Further investigation is required to clarify the effects of enriched-β-conglycinin soybean on the body composition of obese subjects.

Our clinical trial included healthy subjects without a recent history of gastrointestinal disorders, severe acute or chronic diseases, and/or current use of any medication. Therefore, we need to investigate the effectiveness of enriched-β-conglycinin to different types of subjects.

In conclusion, the results of this study revealed that enriched-β-conglycinin soybean has a potential effect on decreasing the serum TG level. Further studies, such as those to determine the biological mechanism for this result, are required to confirm the results of this study. It is important to research and develop food with a rich content of nutrients and functional components, and the present study suggests that the development of new applications of processed foods using enriched-β-conglycinin soybean may help in prevention of lifestyle-related diseases, such as those associated with high serum TG.

## Figures and Tables

**Table 1:** Composition of the enriched-β-conglycinin soybean *Nanahomare* and placebo soybean *Nagomimaru*.

	Enriched-β-conglycinin Soybean Containing Food (Test Meal)			Low-β-conglycinin Soybean Containing Food (Placebo Meal)		
	Flakes (21 g)	Soy Milk (200 mL)	Steamed Soybeans (42 g)	Flakes (21 g)	Soy Milk (200 mL)	Steamed Soybeans (42 g)
Calories (kcal)	89.5	110	74	89.5	112	74
Water (g)	1.7	176.8	24.6	1.7	176.6	24.6
Proteins (g)	7.5	8.8	6.7	7.5	9.6	6.7
Lipids (g)	4.6	6.0	3.9	4.6	6.0	3.9
Carbohydrates (g)	6.2	7.0	2.2	6.2	6.8	2.2
Ash (g)	1.0	1.4	1	1.0	1.2	1
Sodium (mg)	1.0	1.6	105	1.0	2.2	105
β-conglycinin (g)	3.44	4.04	0.66	0.11	0.62	0.12

**Table 2:** Characteristics of the subjects in the test (enriched-β-conglycinin soybean) and placebo groups.

Characteristic	Test Group	Placebo Group	*p*
Subjects, *n*	70	64	–
Males, *n* (%)	21 (30.00%)	20 (31.25%)	0.875
Age, years	52.84 ± 10.18	52.17 ± 10.45	0.707
Height, cm	160.17 ± 8.37	160.59 ± 7.56	0.763
Body weight, kg	57.43 ± 12.11	59.53 ± 11.96	0.316
Body mass index, kg/m^2^	22.28 ± 3.90	22.98 ± 3.67	0.288
Body fat ratio, %	28.07 ± 7.62	29.38 ± 7.21	0.307
Triglyceride, mg/dL	112.81 ± 43.09	113.72 ± 47.42	0.908

Values shown are mean ± standard deviation. Student’s *t*-test was used to compare age, height, body weight, BMI, body fat ratio, and triglyceride between the groups, and the chi-square test was used for gender. *n* = number of subjects.

**Table 3:** Lipid metabolism parameters, blood glucose metabolism parameters, and body composition.

		Value				Change in Value			Time × Group, *p*
		Week 0	Week 4	Week 8	Week 12	From Baseline to Week 4	From Baseline to Week 8	From Baseline to Week 12	
TG in all subjects (mg/dL)	Test	129.04 ± 67.37	108.73 ± 42.97	112.27 ± 51.86	107.49 ± 53.34	−20.31 ± 43.74	−16.77 ± 47.75	−21.30 ± 46.21	0.063
	Placebo	120.72 ± 47.88	120.98 ± 59.60	109.38 ± 48.58	120.58 ± 70.58	0.27 ± 44.13	−11.34 ± 36.81	−0.14 ± 65.83	
	*p*					0.035*	0.466	0.041*	
TG in lower-TG subjects (TG < 100 mg/dL at baseline) (mg/dL)	Test	86.12 ± 27.48	79.52 ± 20.88	78.45 ± 23.43	75.72 ± 23.23	−6.61 ± 28.79	−7.67 ± 28.00	−8.53 ± 30.27	0.667
	Placebo	91.87 ± 34.49	85.06 ± 22.71	81.35 ± 27.46	83.48 ± 23.40	−6.81 ± 28.79	−10.52 ± 36.15	−8.39 ± 27.11	
	*p*					0.721	0.893	0.587	
TG in higher-TG subjects (TG ≥ 100 mg/dL at baseline) (mg/dL)	Test	167.32 ± 69.51	134.78 ± 40.88	142.43 ± 51.80	134.97 ± 56.88	−32.54 ± 51.03	−24.89 ± 59.40	−32.35 ± 54.53	0.075
	Placebo	147.82 ± 42.82	154.73 ± 63.91	135.70 ± 49.64	155.42 ± 81.91	6.91 ± 54.44	−12.12 ± 37.97	7.61 ± 87.83	
	*p*					0.010*	0.757	0.030*	
TC (mg/dL)	Test	219.77 ± 37.49	215.47 ± 36.63	220.66 ± 35.16	222.04 ± 36.45	−4.30 ± 22.86	0.89 ± 17.89	1.81 ± 22.19	0.409
	Placebo	228.00 ± 36.71	226.91 ± 37.31	226.97 ± 36.55	230.23 ± 35.64	−1.09 ± 20.82	−1.03 ± 18.60	2.23 ± 20.87	
	*p*					0.399	0.544	0.910	
LDL-C (mg/dL)	Test	138.66 ± 33.26	138.31 ± 36.56	138.39 ± 33.06	139.94 ± 32.29	−0.34 ± 21.07	−0.27 ± 16.62	0.78 ± 18.45	0.746
	Placebo	146.09 ± 37.51	146.52 ± 38.28	144.13 ± 35.29	145.83 ± 34.83	0.42 ± 19.70	−1.97 ± 18.13	−0.27 ± 19.70	
	*p*					0.829	0.573	0.752	
HDL-C (mg/dL)	Test	66.86 ± 16.41	68.19 ± 17.36	66.26 ± 18.12	68.67 ± 18.73	1.33 ± 7.49	−0.60 ± 6.43	1.80 ± 7.75	0.276
	Placebo	68.45 ± 17.21	69.19 ± 17.21	67.83 ± 17.81	68.42 ± 17.78	0.73 ± 6.39	−0.63 ± 6.36	−0.03 ± 6.56	
	*p*					0.624	0.982	0.146	
FPG (mg/dL)	Test	88.67 ± 16.41	88.84 ± 17.36	89.84 ± 18.12	89.67 ± 18.73	0.17 ± 5.02	1.17 ± 6.64	0.93 ± 6.54	0.013 *
	Placebo	88.69 ± 17.21	89.52 ± 17.21	88.03 ± 17.81	88.05 ± 17.78	0.83 ± 4.14	−0.66 ± 4.15	−0.64 ± 5.48	
	*p*					0.413	0.061	0.138	
HbA1c (%)	Test	5.34 ± 0.43	5.39 ± 0.41	5.35 ± 0.42	5.30 ± 0.40	0.05 ± 0.13	0.01 ± 0.20	−0.05 ± 0.17	0.104
	Placebo	5.36 ± 0.40	5.42 ± 0.39	5.37 ± 0.37	5.28 ± 0.40	0.06 ± 0.12	0.01 ± 0.15	−0.08 ± 0.17	
	*p*					0.526	0.907	0.283	
Insulin (μU/mL)	Test	4.23 ± 4.09	4.40 ± 4.32	3.71 ± 2.76	3.78 ± 2.93	0.17 ± 2.27	−0.52 ± 2.45	−0.47 ± 2.79	0.155
	Placebo	4.08 ± 2.18	4.61 ± 2.83	3.69 ± 2.51	3.85 ± 2.08	0.54 ± 1.90	−0.38 ± 1.67	−0.22 ± 1.60	
	*p*					0.317	0.717	0.542	
BW (kg)	Test	57.43 ± 12.00	57.80 ± 12.03	57.75 ± 12.06	57.81 ± 12.00	0.37 ± 0.73	0.32 ± 0.91	0.32 ± 1.24	0.037 *
	Placebo	59.52 ± 12.10	59.80 ± 12.15	59.98 ± 12.23	60.12 ± 12.53	0.28 ± 0.76	0.46 ± 0.96	0.59 ± 1.13	
	*p*					0.477	0.405	0.184	
BFR (%)	Test	28.27 ± 7.62	28.63 ± 7.66	28.53 ± 7.72	28.59 ± 7.82	0.37 ± 1.06	0.27 ± 1.33	0.38 ± 1.61	0.443
	Placebo	29.59 ± 7.34	30.01 ± 7.14	30.21 ± 7.18	30.19 ± 7.57	0.42 ± 1.66	0.62 ± 1.36	0.60 ± 1.81	
	*p*					0.834	0.133	0.453	
BMI (kg/m^2^)	Test	22.29 ± 3.86	22.43 ± 3.85	22.42 ± 3.89	22.44 ± 3.89	0.14 ± 0.30	0.13 ± 0.35	0.13 ± 0.48	0.054
	Placebo	22.99 ± 3.75	23.09 ± 3.73	23.17 ± 3.77	23.22 ± 3.89	0.10 ± 0.30	0.18 ± 0.36	0.22 ± 0.42	
	*p*					0.444	0.449	0.242	

TG, triglyceride; TC, total cholesterol; LDL-C, low-density lipoprotein cholesterol; HDL-C, high density lipoprotein cholesterol; FPG, fasting plasma glucose; HbA1c, hemoglobin A1c; BW, body weight; BFR, body fat ratio; BMI, body mass index. Values shown are mean ± standard deviation. Changes in subject values were analyzed using repeated measures ANOVA between the groups. Changes in subject values were analyzed using Student’s *t*-test to compare the mean of the test food group and the placebo food group at each evaluation point. * Statistically significant, *p* < 0.05.

**Table 4:** Improvement frequencies of lipid metabolism parameters, blood glucose metabolism parameters, and body composition.

		Improved Subjects *n (%)*	Unimproved Subjects *n (%)*	*p*
TG in all subjects	Test	47 (67%)	23 (33%)	0.265
	Placebo	37 (58%)	27 (42%)	
TG in lower-TG subjects (TG < 100 mg/dL at baseline)	Test	19 (58%)	14 (42%)	0.762
	Placebo	19 (61%)	12 (39%)	
TG in higher-TG subjects (TG ≥ 100 mg/dL at baseline)	Test	28 (76%)	9 (24%)	0.063
	Placebo	18 (55%)	15 (45%)	
TC	Test	32 (46%)	38 (54%)	0.336
	Placebo	24 (38%)	40 (63%)	
LDL-C	Test	33 (47%)	37 (53%)	0.975
	Placebo	30 (47%)	34 (53%)	
HDL-C	Test	41 (59%)	29 (41%)	0.086
	Placebo	28 (44%)	36 (56%)	
FPG	Test	28 (40%)	42 (60%)	0.245
	Placebo	32 (50%)	32 (50%)	
HbA1c	Test	32 (46%)	38 (54%)	0.021 *
	Placebo	42 (66%)	22 (34%)	
Insulin	Test	40 (57%)	30 (43%)	0.235
	Placebo	30 (47%)	34 (53%)	
BW	Test	22 (31%)	48 (69%)	0.982
	Placebo	20 (31%)	44 (69%)	
BFR	Test	24 (34%)	46 (66%)	0.241
	Placebo	16 (25%)	48 (75%)	
BMI	Test	20 (29%)	50 (71%)	0.795
	Placebo	17 (27%)	47 (73%)	

At week 12 after the beginning of ingestion. Improvement frequencies were analyzed by chi-square test. *n* = number of subjects. * Statistically significant, *p* < 0.05.

**Table 5:** Biochemical data.

		Week 0	Week 4	Week 8	Week 12
WBC (10^3^/μL)	Test	5.70 ± 1.35	5.78 ± 1.56	5.68 ± 1.42	5.73 ± 1.45
	Placebo	5.71 ± 1.38	5.81 ± 1.63	5.75 ± 1.37	5.73 ± 1.58
RBC (10^4^/μL)	Test	462.07 ± 45.34	461.37 ± 39.93	463.09 ± 42.31	470.78 ± 46.36
	Placebo	466.52 ± 41.84	462.52 ± 41.26	463.56 ± 40.05	469.14 ± 40.70
Hb (g/dL)	Test	13.94 ± 1.69	13.97 ± 1.66	13.96 ± 1.72	14.22 ± 1.89
	Placebo	14.20 ± 1.62	14.06 ± 1.59	14.02 ± 1.57	14.23 ± 1.60
Ht (%)	Test	42.60 ± 4.40	42.55 ± 4.20	42.64 ± 4.34	43.62 ± 4.99
	Placebo	42.98 ± 4.14	42.55 ± 4.07	42.68 ± 4.02	43.43 ± 4.03
Plt (10^4^/μL)	Test	25.19 ± 6.77	25.94 ± 6.33	25.67 ± 7.12	25.72 ± 7.01
	Placebo	25.26 ± 5.23	25.43 ± 5.87	25.46 ± 5.51	25.65 ± 5.81
AST (U/L)	Test	24.21 ± 9.33	23.51 ± 7.99	23.23 ± 7.46	23.09 ± 6.15
	Placebo	23.36 ± 8.64	23.61 ± 8.27	24.23 ± 7.61	23.86 ± 7.70
ALT (U/L)	Test	24.07 ± 15.33	23.97 ± 14.76	22.41 ± 12.97	21.20 ± 11.40
	Placebo	22.63 ± 14.38	25.48 ± 17.26	24.52 ± 18.06	22.94 ± 16.08
γ-GTP (U/L)	Test	30.99 ± 22.99	32.4 ± 29.89	28.09 ± 18.48	27.65 ± 17.29
	Placebo	32.06 ± 31.68	40.02 ± 73.19	34.19 ± 44.79	30.84 ± 27.97
ALP (U/L)	Test	215.97 ± 63.40	216.16 ± 70.10	209.94 ± 59.94	215.72 ± 64.00
	Placebo	214.16 ± 74.31	219.36 ± 79.55	219.5 ± 93.23	218.77 ± 82.34
LDH (U/L)	Test	203.47 ± 35.03	206.91 ± 33.63	207.76 ± 32.24	211.68 ± 34.94
	Placebo	202.72 ± 25.98	202.97 ± 25.08	208.16 ± 25.15	208.92 ± 26.68
BUN (mg/dL)	Test	12.73 ± 3.14	14.05 ± 3.53	13.94 ± 3.63	14.10 ± 3.45
	Placebo	12.95 ± 3.28	13.70 ± 3.10	13.91 ± 3.30	13.68 ± 3.62
CRE (mg/dL)	Test	0.73 ± 0.14	0.74 ± 0.14	0.74 ± 0.14	0.74 ± 0.15
	Placebo	0.74 ± 0.14	0.74 ± 0.13	0.74 ± 0.13	0.73 ± 0.14
UA (mg/dL)	Test	5.14 ± 1.5	5.20 ± 1.41	5.08 ± 1.36	5.06 ± 1.32
	Placebo	5.20 ± 1.29	5.25 ± 1.34	5.18 ± 1.21	5.26 ± 1.24
SBP (mmHg)	Test	118.23 ± 19.67	118.2 ± 18.99	117.67 ± 18.86	121.51 ± 19.17
	Placebo	118.11 ± 13.97	116.89 ± 14.51	116.34 ± 15.46	123.3 ± 16.94
DBP (mmHg)	Test	74.45 ± 12.38	73.94 ± 11.28	74.44 ± 10.03	77.33 ± 11.86
	Placebo	74.23 ± 10.19	74.48 ± 10.17	74.08 ± 10.51	78.67 ± 9.93

WBC, white blood cells; RBC, red blood cells; Hb, hemoglobin, Ht, hematocrit; Plt, platelet count; AST, aspartate aminotransferase; ALT, alanine aminotransferase; γ-GTP, gamma glutamyl transpeptidase; ALP, alkaline phosphatase; LDH, lactate dehydrogenase; BUN, blood urea nitrogen; CRE, creatinine; UA, uric acid; SBP, systolic blood pressure; DBP, diastolic blood pressure. Values are mean ± standard deviation.

## Supplementary Materials

The following are available online at http://www.mdpi.com/2072-6643/8/8/491/s1, Figure S1: The correlation between the change in the logarithmically transformed TG values and BMI from baseline to week 12.

## Abbreviations

ALP	alkaline phosphatase
ALT	alanine aminotransferase
AST	aspartate aminotransferase
BFR	body fat ratio
BMI	body mass index
BP	blood pressure
BUN	blood urea nitrogen
BW	body weight
CBC	complete blood count
CHD	coronary heart disease
CRE	creatinine
CVD	cardiovascular disease
FPG	fasting plasma glucose
γ-GTP	gamma glutamyl transpeptidase
HbA1c	hemoglobin A1c
Hb	hemoglobin
HDL-C	high density lipoprotein cholesterol
Ht	hematocrit
LDH	lactate dehydrogenase
LDL-C	low-density lipoprotein cholesterol
Plt	platelet count
RBC	red blood cells
TC	total cholesterol
TG	triglyceride
UA	uric acid
WBC	white blood cells
