# Supplementary material for: Improvement of Triglyceride Levels through the Intake of Enriched-β-Conglycinin Soybean (Nanahomare) Revealed in a Randomized, Double-Blind, Placebo-Controlled Study
Source: Nutrients. 2016 Aug 11;8(8):491. doi: 10.3390/nu8080491 (PMC4997404; doi:10.3390/nu8080491)
Supplement: Supplementary file 1 [file nutrients-08-00491-s001.docx]

Supplementary Materials: Improvement of Triglyceride Levels through the Intake of
Enriched-β-conglycinin Soybean (*Nanahomare*) Revealed in a Randomized, Double-Blind,
Placebo-Controlled Study

Mie Nishimura, Tatsuya Ohkawara, Yuji Sato, Hiroki Satoh, Yoko Takahashi,
Makita Hajika and Jun Nishihira


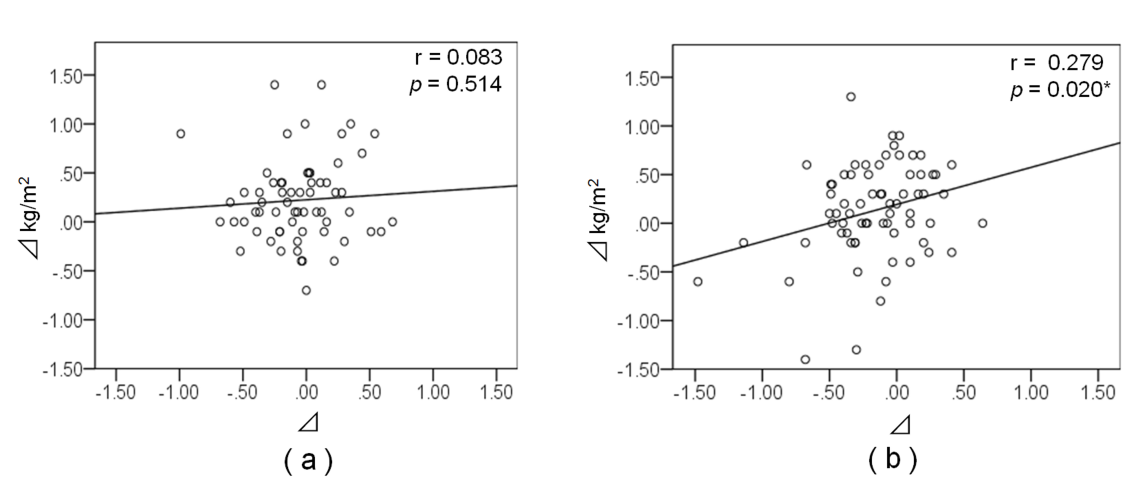


**Figure S1.** The correlation between the change in the logarithmically transformed TG values and BMI from baseline to week 12. (**a**) Placebo group (**b**) test group. Horizontal axis, the change in the logarithmically transformed TG values from baseline to week 12; longitudinal axis, the change in BMI values from baseline to week 12. The strength of the association between the two variables was measured using Pearson’s correlation coefficient (r). * Statistically significant, *p* < 0.05.
